# Combination of quadruplex qPCR and next-generation sequencing for qualitative and quantitative analysis of the HIV-1 latent reservoir

**DOI:** 10.1084/jem.20190896

**Published:** 2019-07-26

**Authors:** Christian Gaebler, Julio C.C. Lorenzi, Thiago Y. Oliveira, Lilian Nogueira, Victor Ramos, Ching-Lan Lu, Joy A. Pai, Pilar Mendoza, Mila Jankovic, Marina Caskey, Michel C. Nussenzweig

**Affiliations:** 1 Laboratory of Molecular Immunology, The Rockefeller University, New York, NY; 2 Howard Hughes Medical Institute, The Rockefeller University, New York, NY

## Abstract

HIV-1 infection requires lifelong therapy with antiretroviral drugs due to the existence of a latent reservoir of transcriptionally inactive integrated proviruses. The goal of HIV-1 cure research is to eliminate or functionally silence this reservoir. To this end, there are numerous ongoing studies to evaluate immunological approaches, including monoclonal antibody therapies. Evaluating the results of these studies requires sensitive and specific measures of the reservoir. Here, we describe a relatively high-throughput combined quantitative PCR (qPCR) and next-generation sequencing method. Four different qPCR probes covering the packaging signal (PS), group-specific antigen (*gag*), polymerase (*pol*), and envelope (*env*) are combined in a single multiplex reaction to detect the HIV-1 genome in limiting dilution samples followed by sequence verification of individual reactions that are positive for combinations of any two of the four probes (Q4PCR). This sensitive and specific approach allows for an unbiased characterization of the HIV-1 latent reservoir.

## Abstract (precis)

HIV-1 cure research seeks to decrease or eliminate the latent reservoir. The evaluation of such curative strategies requires accurate measures of the reservoir. Gaebler et al. describe a combined multicolor qPCR and next-generation sequencing method that enables the sensitive and specific characterization of the HIV-1 latent reservoir.

## Introduction

Like other retroviruses, HIV-1 integrates into the host genome, where it is transcribed to produce infectious virions (Craigie and Bushman, 2012). Productive infection typically leads to cell death; however, in a small number of CD4^+^ T cells, the integrated virus is silenced and becomes latent. Combination antiretroviral therapy (cART) is highly effective in suppressing HIV-1 infection and preventing disease progression; however, cART does not eliminate the virus due to the existence of the latent reservoir (Chun et al., 1997; Finzi et al., 1997; Wong et al., 1997). Longitudinal studies performed on individuals on cART indicate that the latent reservoir has a half-life of 44 mo (Chun et al., 1999; Siliciano et al., 2003). Thus, treatment interruption leads almost invariably to rapid viral rebound, and therapy with cART is required for the lifetime of the infected individual (Margolis and Archin, 2017; Sengupta and Siliciano, 2018).

An important goal for HIV-1 research is to achieve a functional remission or cure by decreasing or eliminating the latent reservoir, and a number of clinical trials have been designed to test new approaches to this problem (Cillo and Mellors, 2016; Margolis et al., 2017; Gruell and Klein, 2018; Caskey et al., 2019). The evaluation of HIV-1 curative strategies requires sensitive, specific, and precise assays to quantify and characterize the latent HIV-1 reservoir. Yet, to date, most approaches show major discrepancies in infected cell frequencies (Eriksson et al., 2013; Sengupta and Siliciano, 2018). These inconsistencies constrain the accurate assessment of HIV-1 cure efforts and could obscure a meaningful intervention (Henrich et al., 2017; Sengupta and Siliciano, 2018).

Here, we report a relatively high-throughput method for enumerating and characterizing intact latent proviral DNA by a combination of multicolor quantitative PCR (qPCR) and next-generation sequencing. We compare the new method to quantitative and qualitative viral outgrowth assays (Q^2^VOAs) and near full-length (NFL) sequencing on paired peripheral blood samples obtained at two time points from the same six individuals enrolled in a clinical trial that involved analytical treatment interruption after infusion of a combination of two broadly neutralizing monoclonal antibodies (Lu et al., 2018; Mendoza et al., 2018).

## Results

### qPCR primers and probes

**Figure 1.**
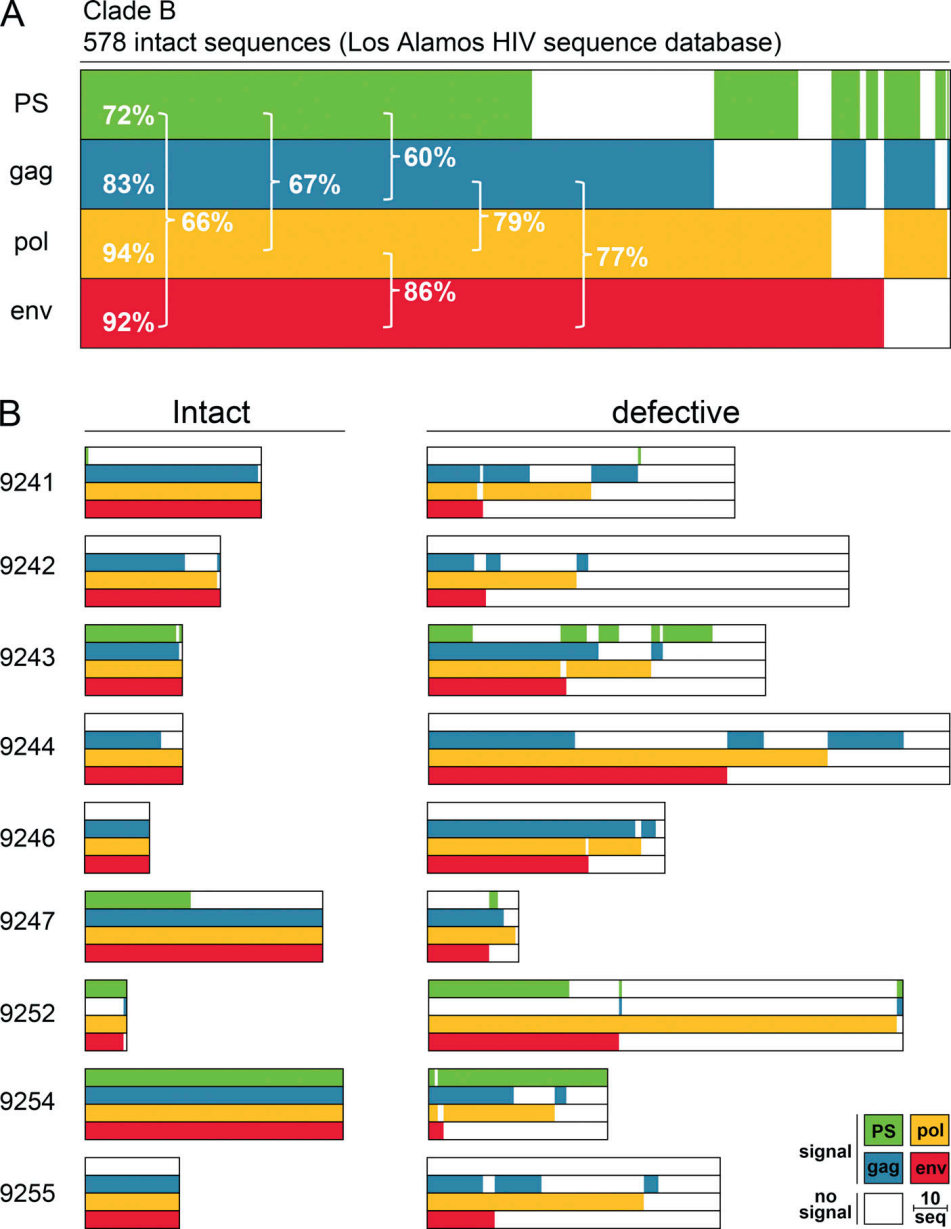
**Predicted detection of HIV-1. (A)** Horizontal bars represent the predicted detection of 578 intact clade B proviral sequences (Los Alamos HIV sequence database) by qPCR primer/probe sets that target PS (green), *gag* (blue), *pol* (yellow), and *env* (red) regions. Signal prediction for each individual proviral sequence is represented by the presence of the color of the respective primer/probe set. Sequences containing polymorphisms that prevent signal detection are represented by the absence of color. The percentage indicates the fraction of detected sequences for individual primer/probe sets or combinations of two primer/probe sets (brackets). **(B)** Horizontal bars represent the predicted detection of 401 intact and 977 defective NFL genomes from nine individuals (Lu et al., 2018; Mendoza et al., 2018). The same primer/probe sets and color scheme are used as described above. The group of defective sequences includes NFL genomes that carry small insertions, deletions, and defects in the packaging site and/or MSD. The length of the scale bar represents 10 proviral sequences.

To select primer/probe sets that maximize detection of HIV-1, we analyzed four previously characterized candidates in silico using intact proviral genomes from the Los Alamos HIV sequence database (Palmer et al., 2003; Schmid et al., 2010; Bruner and Siliciano, 2018; Bruner et al., 2019). The selected primer/probes cover conserved regions in the HIV-1 genome, including the packaging signal (PS), group-specific antigen (*gag*), polymerase (*pol*), and envelope (*env*). Allowing for one and up to four mismatches (three mismatches at the 5′ end and one mismatch at the 3′ end) in probes and primers, respectively, the PS, *gag*, *pol*, and *env* primer/probes detected 72%, 83%, 94%, and 92% of 578 intact clade B sequences in the Los Alamos HIV sequence database (Figs. 1 A and S1; Stadhouders et al., 2010; Lefever et al., 2013; Rutsaert et al., 2018). All genomes scored positive with at least one of the four primer/probe sets. Notably, the large majority (99%) of genomes were positive for at least one of the many combinations of two primer/probe sets. However, any single two-probe combination was at best 86% sensitive (*pol+env*).

To test whether these primer/probe sets can discriminate between intact and defective proviruses, we also performed the same in silico analysis on 1,378 intact and defective HIV-1 sequences from nine individuals that received a combination of two broadly neutralizing monoclonal antibodies during treatment interruption (Lu et al., 2018). In six out of nine patients, we observed HIV-1 sequence polymorphisms that cause a predicted loss of signal for at least one of the primer/probe sets (Fig. 1 B). For example, intact viruses in individuals 9241, 9242, 9244, 9246, and 9255 are predicted to be negative for the PS primer/probe set. In addition to the problem of sensitivity, two probe combinations also have a potential problem with specificity, since a number of defective viruses were predicted to be positive for several of the two probe combinations tested. The potential magnitude of this problem varies with the probe combination and the individual analyzed. For example, in 9252, of the 61 viruses detected with the PS+*env* combination, 80% are defective (49 defective vs. 12 intact), whereas in 9243, it is 35% (Fig. 1 B). Thus, the in silico data suggest that any single combination of two probes would not be sufficient for sensitive and specific reservoir measurements due to HIV-1 sequence polymorphisms within and between individuals.

### Quadruplex qPCR (Q4PCR)

To accommodate HIV-1 sequence diversity, we developed a multiplex qPCR strategy for simultaneous detection of four probes: PS, *gag*, *pol*, and *env* (Q4PCR). The new method enables relatively high-throughput measurements of the latent HIV-1 reservoir with real-time detection of DNA amplification, the exclusion of gel electrophoresis, and the use of a 384-well format. Using this approach, we analyzed samples from two separate time points from six individuals enrolled in a clinical trial that involved analytical treatment interruption after infusion of a combination of two broadly neutralizing monoclonal antibodies (Lu et al., 2018; Mendoza et al., 2018).

**Figure 2.**
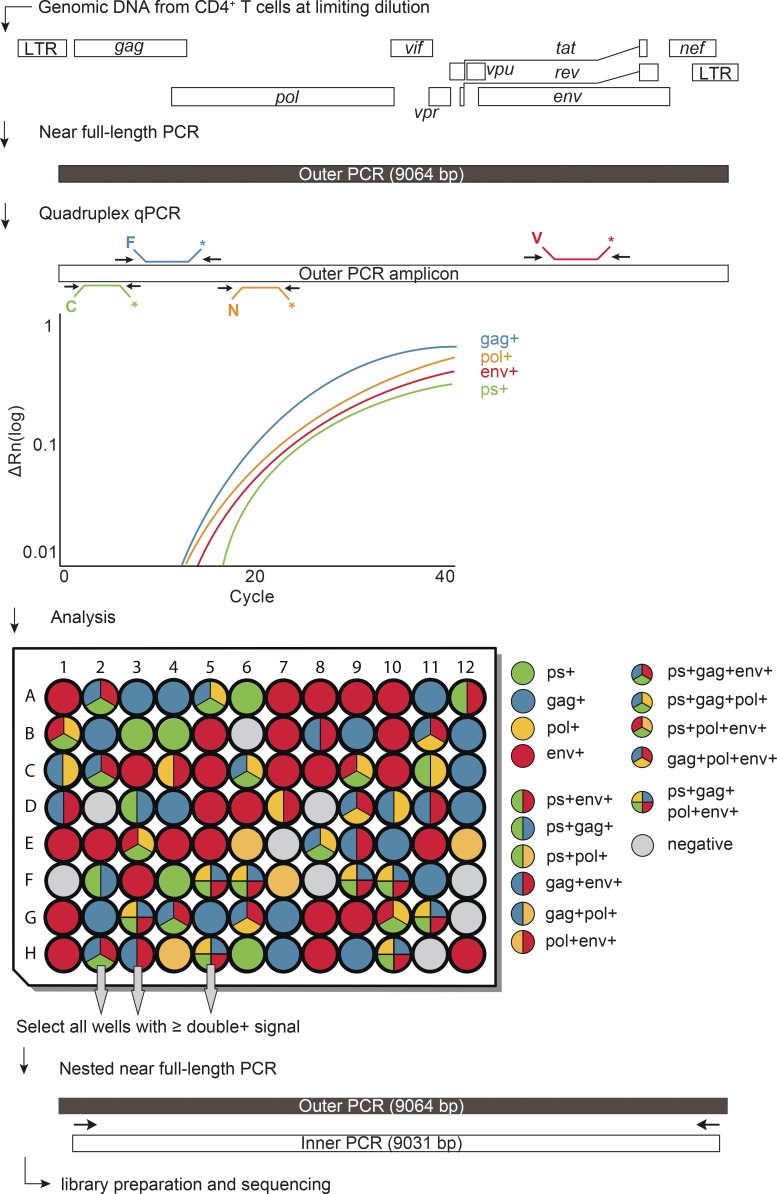
**Q4PCR approach.** Schematic representation of the Q4PCR protocol. Genomic DNA from CD4^+^ T cells was subjected to limiting dilution qPCR with a *gag*-specific primer/probe set to determine overall HIV-1 proviral frequency. NFL proviral genomes were amplified from CD4^+^ T cell genomic DNA in samples diluted to single-copy concentrations based on *gag* qPCR. An aliquot of the resulting amplicons was assayed by Q4PCR using a combination of primer/probe sets covering PS, *gag*, *pol*, and *en*v. Samples with positive signal for any combination of at least two primer/probe sets were collected and subjected to nested NFL PCR, library preparation, and next-generation sequencing.

Proviral genomes were amplified from DNA extracted from purified CD4^+^ T cells obtained 2 wk before and 12 wk after treatment interruption. To determine overall HIV-1 proviral frequency, genomic DNA from CD4^+^ T cells was assayed for *gag* by qPCR. We found *gag*^+^ proviruses with the expected variation between individuals at a median frequency of 417 out of 10^6^ CD4^+^ T cells (Table S1). DNA from CD4^+^ T cells was diluted to a concentration equivalent to a single copy of *gag* per reaction and assayed by Q4PCR (Fig. 2).

Individual reactions containing a single proviral copy were amplified to produce NFL and subgenomic proviruses using 5′ of *gag* and LTR primers (Li et al., 2007; Ho et al., 2013). Individual amplicons were then tested for reactivity with each of the four qPCR probes. Participant 9254 was excluded from the quantitative analysis because of inadequate sample availability.

### Quantitative analysis

**Figure 3.**
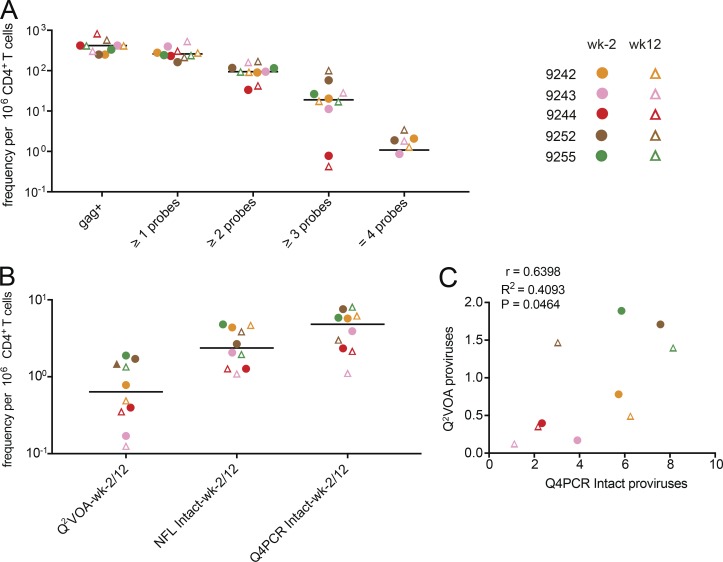
**Quantitative analysis. (A)** Frequency per million CD4^+^ T cells of *gag*^+^ proviruses amplified from genomic DNA and samples with any one, two, three, or all four qPCR probe signals after NFL amplification for the preinfusion (wk−2) and week 12 (wk12) time points. Horizontal bars indicate median values. For patient 9242, the frequency of *env^+^* proviruses amplified from genomic DNA per million CD4^+^ T cells is plotted due to limited *gag^+^* amplification signal. **(B)** Comparison of frequencies of inducible proviruses (Q^2^VOA), intact proviruses obtained with NFL sequencing strategy (NFL intact), and intact proviruses identified with Q4PCR (Q4PCR intact) at preinfusion and week 12 time points for the same samples (Lu et al., 2018; Mendoza et al., 2018). **(C)** Pearson correlation between frequency of intact proviruses identified with Q4PCR and inducible proviruses measured by Q^2^VOA at preinfusion and week 12 time points (Lu et al., 2018; Mendoza et al., 2018). Participant 9254 was excluded from the quantitative analysis because of inadequate sample availability. Individual patients are depicted in different colors. Time points are represented by circles and triangles for week −2 and week 12, respectively.

The number of samples showing reactivity with any one of the selected HIV-1 probes after NFL amplification was similar (1.5-fold lower) to the number predicted from short segment *gag* qPCR performed before the amplification indicating that the NFL genome PCR reaction was efficient (Fig. 3 A and Table S1).

There was substantial variation between individuals (see Fig. 6 and Sensitivity and predictive value), but the median frequency of proviruses reactive to two or more probes was 94 × 10^−6^ CD4^+^ T cells or ∼4× less than *gag* DNA alone. The frequency of proviruses that scored positive with at least three or all four probes was lower still at 19 × 10^−6^ and 1 × 10^−6^ CD4^+^ T cells (Fig. 3 A and Table S1).

To determine which of the proviruses detected by Q4PCR were intact, we performed next-generation sequencing (Fig. 2). Preliminary sequence analysis indicated that samples reacting with only a single primer/probe or only the PS+*gag* combination were defective, and these samples were mostly omitted from further analysis. All other samples showing reactivity with two or more of the four qPCR probes were sequenced.

1,832 proviruses were sequenced. On average, we obtained 153 proviral sequences per time point per participant. Proviral sequences were scored as intact if they did not have deletions or insertions, were in frame, did not contain stop codons, and had intact packaging signals and major splice donors (MSDs; Fig. S2). In total, we found 237 intact and 1,595 defective proviral sequences. Intact proviruses were found at a median frequency of 4.8 × 10^−6^ CD4^+^ T cells, a nearly 20-fold lower frequency than proviruses reacting with two or more qPCR probes (Fig. 3, A and B). NFL sequencing and Q^2^VOA performed on the same samples showed 2-fold and 7.6-fold fewer intact and inducible proviruses per million CD4^+^ T cells than Q4PCR, respectively (Lu et al., 2018; Mendoza et al., 2018; Fig. 3 B and Table S2). Despite the limited sample size, there was a significant correlation between the number of viruses measured by outgrowth assays and Q4PCR (Fig. 3 C). Furthermore, the detection of intact proviruses by Q4PCR was highly reproducible. In two sets of independent experiments performed on samples from four individuals, there was a strong agreement (Pearson r = 0.7764, P = 0.0235) consistent with 1.5-fold variability in the assay (Fig. S3 A). This degree of variation matches Q^2^VOA (clone frequency in repeat experiments, R^2^ = 0.85; Cohen et al., 2018) and compares favorably with NFL sequencing (2.5-fold variability; Lu et al., 2018).

### Comparison with Q^2^VOA and NFL sequencing

**Figure 4.**
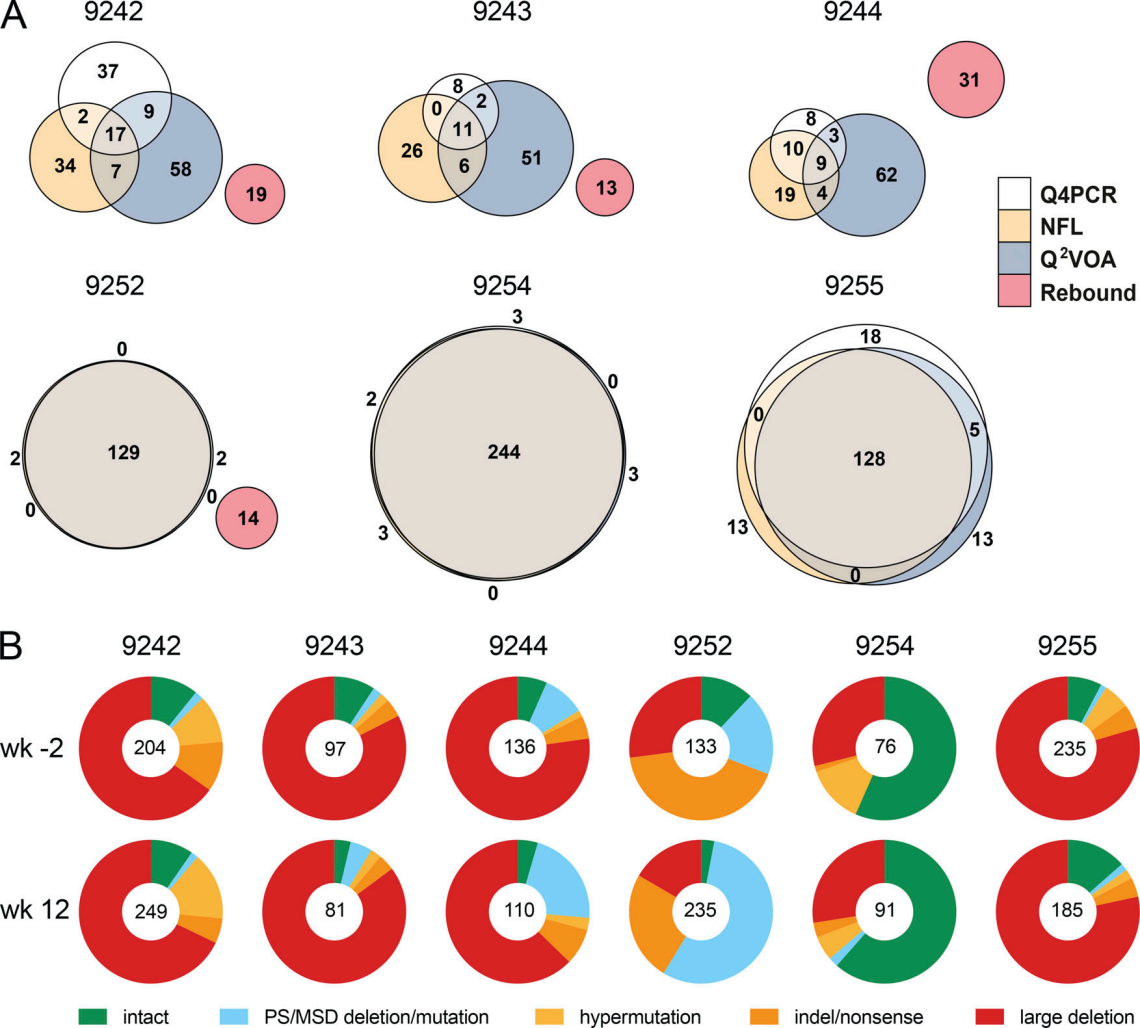
**Qualitative sequence analysis. (A)** Euler diagrams representing the overlap between *env* sequences obtained from Q4PCR (white), Q^2^VOA (blue), NFL sequencing (yellow), and rebound plasma SGA or PBMC outgrowth culture (red) from participants 9242, 9243, 9244, 9252, 9254, and 9255 (Lu et al., 2018; Mendoza et al., 2018). Q4PCR, Q^2^VOA, and NFL sequences obtained from the preinfusion and week 12 time point were combined. Identical *env* sequences were considered as shared sequences. The number inside overlapping areas is the sum of all shared sequences. **(B)** Pie charts depict the distribution of intact and defective proviral sequences at the preinfusion (wk−2) and week 12 (wk12) time points. The number in the middle of the pie represents the total number of proviruses sequenced. Pie slices indicate the proportion of sequences that were intact or had different defects, including PS defects and MSD site mutations (blue), premature stop codons mediated by hypermutation (yellow), single-nucleotide indels or nonsense mutations (orange), and sequences with large internal deletions (red).

To compare the intact proviruses obtained by Q4PCR with those obtained by Q^2^VOA and NFL sequencing, we created Euler diagrams and phylogenetic trees using *env* sequences from preinfusion and week 12 time points combined (Lu et al., 2018; Mendoza et al., 2018; Fig. 4 A, Fig. S4, and Table S3). There was a substantial overlap between *env* sequences identified by all three methods. As would be expected, the greatest overlap was found in individuals whose reservoir was dominated by expanded clones (9252, 9254, and 9255), and the least in those with a more diverse latent reservoir (9242, 9243, and 9244; Fig. 4 A). Overall, 68% and 66% of intact Q4PCR sequences were identical to intact NFL and Q^2^VOA sequences, respectively. Thus, we have been unable to detect a selection bias in the HIV-1 reservoir sequences obtained by Q4PCR compared with Q^2^VOA or NFL sequencing. Overall, we observed that the total number of sequences identified with each assay was related to the number of CD4^+^ T cells assayed (Fig. S3 B and Table S2).

In line with our previous observations from both Q^2^VOA and NFL sequencing, the new Q4PCR sequencing strategy was unable to detect a sequence match between intact proviral sequences and rebound viruses obtained by single genome analysis (SGA) at the time of rebound (Lu et al., 2018).

### Qualitative analysis

To determine how integrated HIV-1 varied between individuals and time points within an individual we analyzed both defective and intact proviruses (Fig. 4 B and Table S4). Defective viruses were further divided into those with defective packaging signals or MSDs, hypermutation, indels or nonsense mutations, and large deletions. The distribution of defective and intact viruses was relatively constant between the two time points in each individual, with the exception of 9252, who showed a relative decrease in the number of intact proviruses and an increased representation of proviruses with defective packaging signals or MSDs between weeks −2 and 12. Overall, large internal deletions were the most frequent source of defective proviruses followed by packaging site and/or MSD defects, indel/nonsense mutations, and hypermutation. However, the contribution of intact proviruses and individual categories of defects varied significantly between individuals. For example, when the two time points are combined, the fraction of intact proviruses in individual 9244 was only 5.7% compared with 59% for 9254.

### Sensitivity and predictive value

To examine individual probes and probe combinations for their ability to identify intact proviruses, we compared sequencing results with qPCR and determined the predictive value of each probe. The positive predictive value is the probability that a sample that is reactive with a specific probe or combination of probes is associated with an intact proviral sequence.

**Figure 5.**
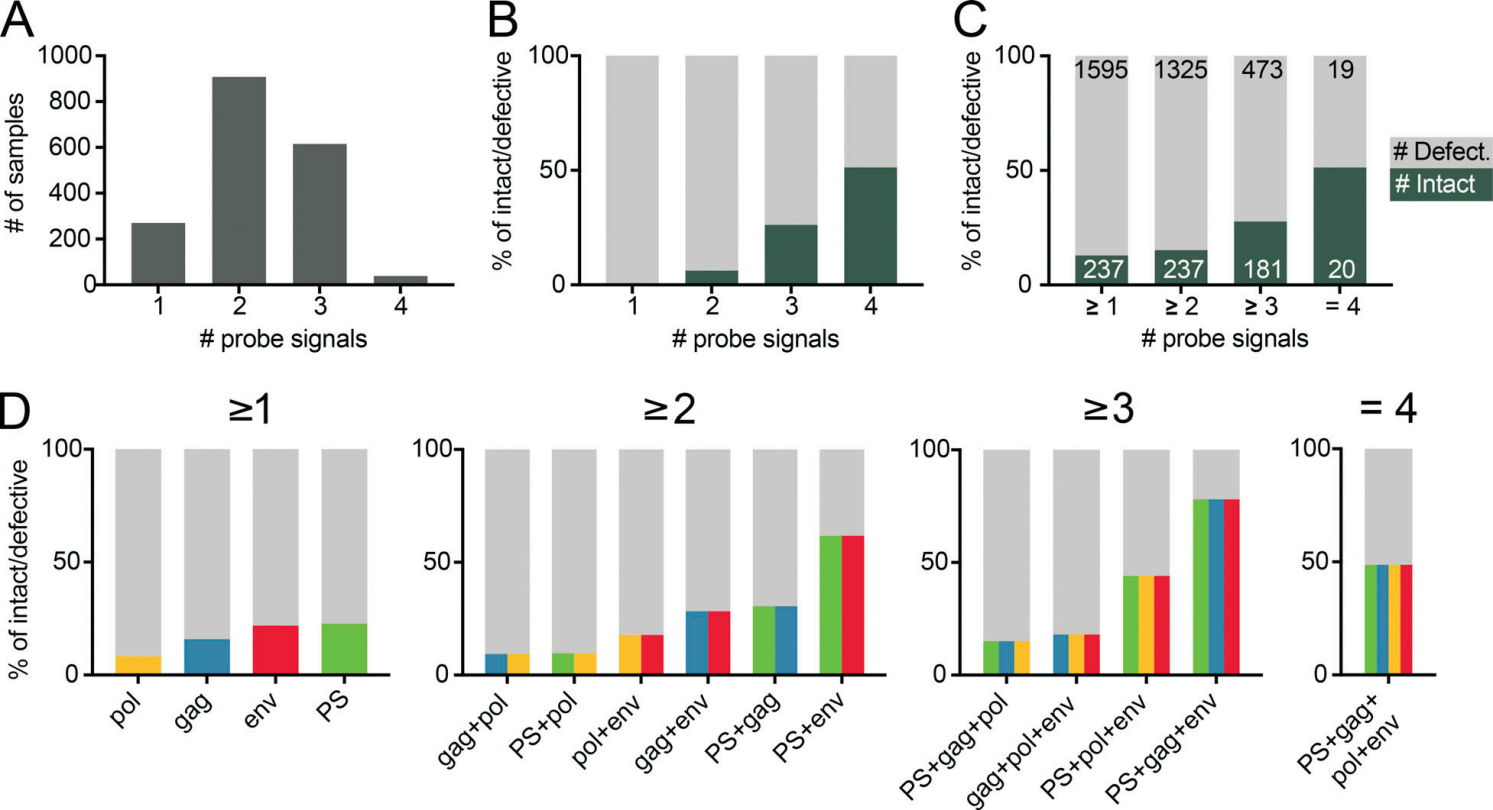
**Probe analysis. (A)** Bar graphs showing the total number of sequenced samples that scored positive for any one, two, three, or all four qPCR probes out of a total of 1,832 assayed. **(B)** Stacked bar graphs showing the predictive value for intact (dark green) and defective (gray) proviral genomes of sequenced samples positive for any one, two, three, or four qPCR probes, respectively. **(C)** Stacked bar graphs showing the predictive value for intact (dark green) and defective (light gray) proviral genomes of sequenced samples positive for at least one, two, three, or four qPCR probes, respectively. The number of samples is depicted in white (intact) or black (defective), respectively. **(D)** Graphs showing the predictive value for intact and defective proviruses of individual probes and all possible combinations of at least two, three, or all four probes, respectively. The predictive value for intact proviruses is colored for each individual probe (PS, green; *gag*, blue; *pol*, yellow; and *env*, red) or as color combinations for specific probe combinations. The defective fraction is shown in gray.

Of a total of 1,832 samples assayed, the majority were positive for only two (*n* = 908) followed by three (*n* = 615), one (*n* = 270), and four (*n* = 39) probe signals (Fig. 5 A). Notably, no sample with an isolated single probe signal was associated with an intact proviral sequence, and only 6% of all samples that were positive for any combination of two probes contained intact proviruses. In contrast, 26% and 51% of all samples that were reactive with any combination of three or four probes, respectively, contained intact proviruses. Thus, the positive predictive value is directly correlated to the number of positive probes (Fig. 5, B and C).

To determine the contribution of combinations of individual probes, we determined the sensitivity and positive predictive value for all possible probe combinations. Notably, whereas an isolated *env* signal fails to predict intact proviruses, *env* in combination with any other probe shows a very high sensitivity for the detection of intact proviruses (98% of 237 intact sequences were *env^+^*). In contrast, the sensitivity of *pol*, PS, or *gag* with any other probe was lower (39%, 65%, and 83%, respectively; Table S5).

**Figure 6.**
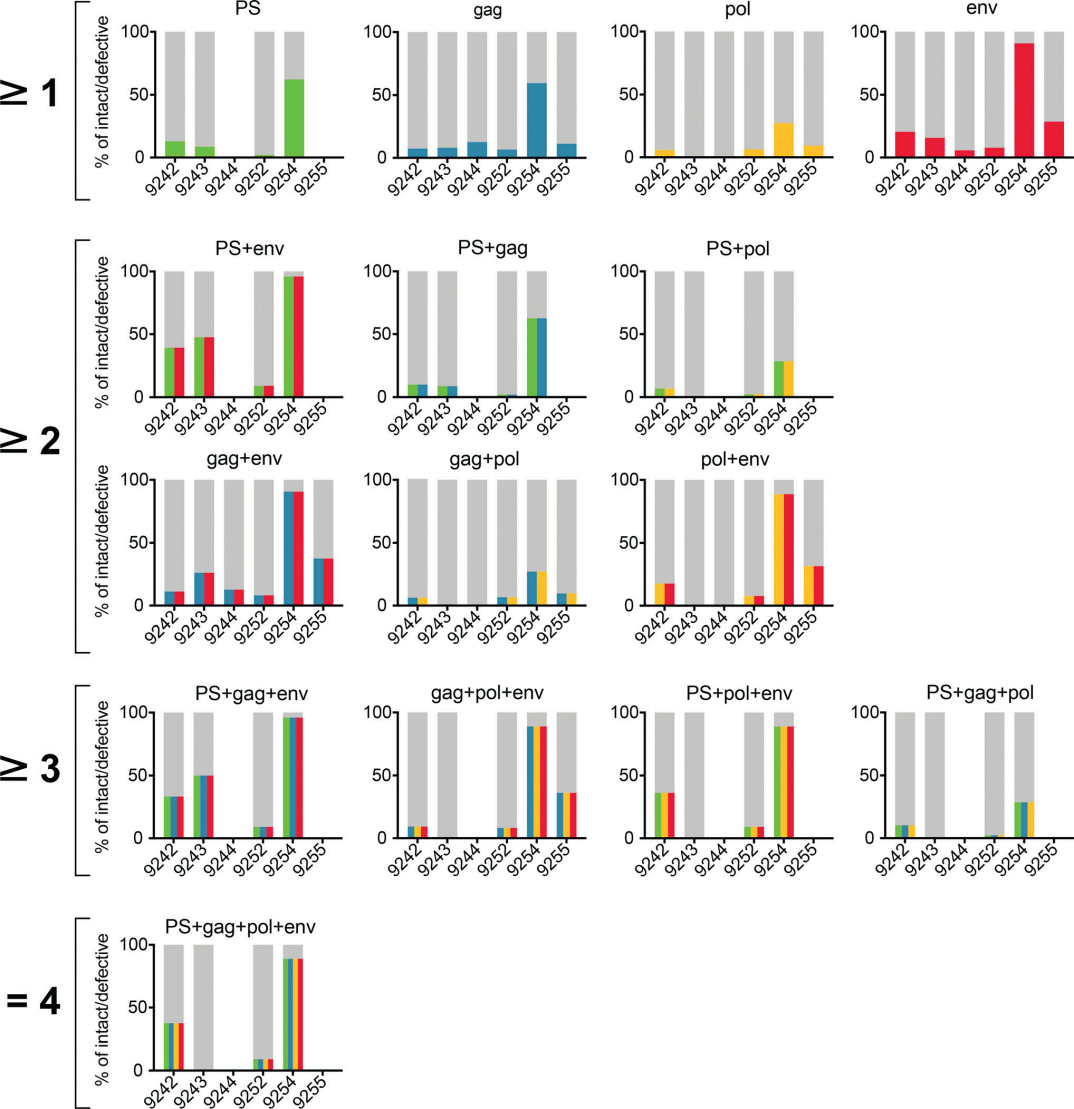
**Probe analysis for individual participants.** Graphs showing the predictive value for intact and defective proviral genomes of individual probes and all possible combinations of at least two, three, or all four probes among participants 9242, 9243, 9244, 9252, 9254, and 9255. The predictive value for intact proviruses is colored for each individual probe (PS, green; *gag*, blue; *pol*, yellow; and *env*, red) or as color combinations for specific probe combinations. The defective fraction is shown in gray. Blank spaces illustrate the absence of specific probe or probe combination signals in individual patients.

The combination of PS+*env* has the highest and *gag+pol* the lowest positive predictive value of any two probe combinations (62% and 9.4%, respectively; Fig. 5 D and Table S5). However, even the PS+*env* combination has a substantial false discovery rate. Overall 38% of PS+*env*-positive samples are associated with defective proviral sequences, and two of the six individuals failed to show any signal with the PS primer/probe set (Fig. 6 and Table S6). In both of these cases, the absence of the signal was predicted and could be explained by sequence polymorphisms in proviral genomes (Fig. 1 B and Fig. S5). Thus, polymorphisms limit the applicability of a diagnostic test based on any two probes including PS+*env*. Moreover, even in those individuals where the PS+*env* combination was effective, the positive predictive value varied from 9.1% to 96% between individuals (Fig. 6 and Table S6).

Samples positive for at least three probes show considerable improved predictive values. For example, samples that were positive for at least PS+*gag*+*env* predict intact proviruses at a rate of 78% (Fig. 5 D). However, the requirement for hybridization with a third or fourth probe decreases the sensitivity of the assay and results in inability to detect 24% and 92% of all positive samples respectively, an effect that appears to be due to HIV-1 sequence variation (Table S5). Therefore, screening with all four primer/probe sets and subsequent sequencing of viruses positive for the combination of any two Q4PCR primer/probe sets is both sensitive and specific for enumeration of intact proviral genomes.

## Discussion

Accurate measurement of the size of the circulating latent reservoir is essential for evaluating therapeutic interventions that aim to eliminate it (Sengupta and Siliciano, 2018). The combination of Q4PCR and next-generation sequencing is a relatively high-throughput method that is both sensitive and specific.

Several methods have been used to evaluate the latent HIV-1 reservoir. For example, total integrated proviral DNA can be measured by PCR using *gag*-specific primers (Bieniasz et al., 1993; Christopherson et al., 2000; Palmer et al., 2003). This assay is simple and quantitative. However, it does not distinguish between rare replication-competent and more abundant replication-defective proviruses (Eriksson et al., 2013; Ho et al., 2013; Bruner et al., 2016). Thus, the overwhelming background of defective proviruses would make any change in the replication competent latent reservoir difficult to detect using this assay. Viral outgrowth assays measure infectious viruses that can be recovered by CD4^+^ T cell activation in vitro (Chun et al., 1997; Lorenzi et al., 2016). However, these assays are very labor intensive, variable, and not sensitive to any change below a factor of 6 (Crooks et al., 2015; Rosenbloom et al., 2019). In addition, these assays selectively underestimate the reservoir because only a fraction of latently infected cells can be induced after a single round of stimulation in vitro (Ho et al., 2013; Hosmane et al., 2017).

Sequencing NFL proviral genomes from limiting dilution CD4^+^ T cell DNA samples allows relatively unbiased characterization of the proviral reservoir (Ho et al., 2013; Hiener et al., 2017; Lu et al., 2018). This technique is very labor intensive and requires interrogation of thousands of reactions per sample making these studies challenging. In addition, there is some variation between the number of estimated intact genomes measured by DNA sequencing assays based on Sanger and next-generation sequencing technologies 12–114 × 10^−6^ (Ho et al., 2013; Bruner et al., 2016) vs. 2.8–24 × 10^−6^ CD4^+^ T cells (Hiener et al., 2017; Lee et al., 2017; Lu et al., 2018; Sharaf et al., 2018; Vibholm et al., 2019). One possible explanation for the observed difference is the use of an empirical Bayesian model to estimate the number of intact proviral genomes in the Sanger-based studies necessitated by the absence of detected intact genomes in 13 out of 26 subjects (Ho et al., 2013; Bruner et al., 2016).

Intact proviral DNA assay (IPDA) is a high-throughput method to measure integrated proviral DNA using droplet digital PCR to probe for the presence of PS+*env* in the proviral genome (Bruner et al., 2019). This method detects intact proviruses at a higher frequency than the sequencing methods (100 × 10^−6^ CD4^+^ T cells; Bruner et al., 2019). The IPDA relies on amplification of two subgenomic regions that together sample 222 bp or only 2% of the 9.7-kb HIV-1 genome. As a result, a significant fraction of proviruses are incorrectly categorized as intact, which leads to an overestimation of intact proviral DNA (Bruner et al., 2019). Moreover, our experiments indicate that polymorphisms lead to variation in the levels of sensitivity and specificity of the PS+*env* probe combination in different individuals suggesting that any single combination of two probes would not be sufficient for broadly applicable reservoir measurements. Most importantly, verification of intact proviruses is not possible in the IPDA, and therefore, the accuracy of this method will vary between individuals depending on the molecular composition of the reservoir. Nevertheless, the IPDA is a high-throughput assay that is more accurate than the total proviral DNA assay, making it the most desirable currently available assay for large studies (Bruner et al., 2019).

The advantage of combining multicolor qPCR and sequencing is that the method is relatively rapid, scalable, and both sensitive and specific. All intact viruses in the six individuals analyzed and 99% of the intact clade B viral sequences in the Los Alamos database were positive for any combination of two of the four probes in the Q4PCR reaction. Bar coding and next-generation sequencing facilitates the analysis of large numbers of samples simultaneously and enables definitive identification of intact proviruses. In addition, the sequence data provide information on the clonal structure of the latent reservoir and on the nature of the defective proviruses.

In conclusion, the combination of four-probe qPCR and next-generation sequencing is a highly sensitive and specific method for measuring intact proviruses in the HIV-1 latent reservoir.

## Materials and methods

### Study participants

HIV-1-infected participants were enrolled at the Rockefeller University Hospital, New York, NY, and the University Hospital Cologne, Cologne, Germany, in an open-label phase 1b study (http://www.clinicaltrials.gov; NCT02825797; EudraCT: 2016–002803-25; Mendoza et al., 2018). All participants provided written informed consent before participation in the study and the study was conducted in accordance with Good Clinical Practice. The protocol was approved by the US Food and Drug Administration, the Paul Ehrlich Institute (Germany), and the institutional review boards at the Rockefeller University and the University of Cologne.

Participants received the combination of two broadly neutralizing antibodies (3BNC117 and 10–1074) intravenously at a dose of 30 mg kg^−1^ body weight of each antibody, at weeks 0, 3, and 6, unless viral rebound occurred. Antiretroviral therapy was discontinued 2 d after the first infusion of antibodies (day 2). Leukapheresis was performed at the Rockefeller University Hospital or at the University Hospital Cologne at week −2 and week 12. Peripheral blood mononuclear cells (PBMCs) were isolated by density gradient centrifugation, and cells were cryopreserved in fetal bovine serum plus 10% DMSO.

### Bioinformatic binding prediction of qPCR primers and probes

Previously characterized primers and probes were mapped to HXB2 to identify regions of binding (Palmer et al., 2003; Malnati et al., 2008; Althaus et al., 2010; Schmid et al., 2010; Bruner and Siliciano, 2018; Bruner et al., 2019). Then, we selected all HIV-1 clade B (*n* = 578), clade C (*n* = 456), and clade A (*n* = 94) full-length intact proviral genomes from the Los Alamos HIV sequence database (https://www.hiv.lanl.gov/content/sequence/NEWALIGN/align.html) that included sequence information for the PS. After alignment to the HXB2 reference sequence using MAFFT v.7.309, we predicted binding and amplification signal for primer/probe sets with a maximum of one probe mismatch and four primer mismatches. Forward and reverse primers were further subdivided in a 5′ and 3′ end, and a maximum of three mismatches at the 5′ end and one mismatch at the 3′ end was allowed for predicted binding and positive signal (Stadhouders et al., 2010; Lefever et al., 2013; Rutsaert et al., 2018). In addition, we used Geneious 11 and Primer3 to assess physical properties such as melting temperatures and secondary structures. Based on predicted binding and favorable physical properties for multiplex qPCR reactions, we selected the PS (Bruner and Siliciano, 2018), *gag* (Palmer et al., 2003), *pol* (Schmid et al., 2010), and *env* (Bruner et al., 2019) primer/probe sets. To test whether these primer/probe sets can discriminate between intact and defective proviruses we analyzed 1,378 intact and defective NFL HIV-1 sequences from nine individuals (Lu et al., 2018).

### CD4^+^ T cell isolation

Total CD4^+^ T cells were isolated from cryopreserved PBMCs by manual magnetic labeling and negative selection using the CD4^+^ T Cell Isolation Kit (Miltenyi Biotec).

### DNA isolation and quantification

Genomic DNA from 1–10 million CD4^+^ T cells was isolated using the Gentra Puregene Cell Kit (Qiagen). In some experiments, DNA was isolated using phenol-chloroform (Klein et al., 2011). Briefly, CD4^+^ T cells were lysed in Proteinase K buffer (100 mM Tris, pH 8, 0.2% SDS, 200 mM NaCl, and 5 mM EDTA) and 20 mg/ml Proteinase K at 56°C for 12 h followed by genomic DNA extraction with phenol/chloroform/isoamyl alcohol extraction and ethanol precipitation. The Qubit 3.0 Fluorometer and Qubit dsDNA BR Assay Kit (Thermo Fisher Scientific) was used to measure DNA concentrations.

### Limiting dilution *gag* qPCR

Genomic DNA was assayed in a 384-well plate format using the Applied Biosystem QuantStudio 6 Flex Real-Time PCR System. HIV-1–specific primers and a probe targeting a conserved region in *gag* were used in a limiting dilution qPCR reaction (forward primer, 5′-ATG​TTT​TCA​GCA​TTA​TCA​GAA​GGA-3′; internal probe, 5′-/6-FAM/CCACCCCAC/ZEN/AAGATTTAAACACCATGCTAA/3′/IABkFQ/; reverse primer, 5′-TGC​TTG​ATG​TCC​CCC​CAC​T-3′; Integrated DNA Technologies; Palmer et al., 2003).

Each qPCR reaction was performed in a 10 µl total reaction volume containing 5 µl of TaqMan Universal PCR Master Mix containing Rox (catalog no. 4304437; Applied Biosystems), 1 µl of diluted genomic DNA, nuclease free water, and the following primer and probe concentrations: 337.5 nM of forward and reverse primers with 93.75 nm of *gag* internal probe. *gag* qPCR conditions were 94°C for 10 min, 50 cycles of 94°C for 15 s, and 60°C for 60 s.

Genomic DNA was serially diluted to concentrations ranging from 2,000 to 250 CD4^+^ T cells per microliter with a minimum of 24 reactions per concentration. We selected DNA dilutions wherein <30% of the *gag* PCR reactions were positive for further analysis because they have a >80% probability of containing a single copy of HIV-1 DNA in each PCR reaction based on the Poisson distribution (Lu et al., 2018).

### NFL HIV-1 PCR (1.PCR)

We used a two-step nested PCR approach to amplify NFL HIV-1 genomes. All reactions were performed in a 20 µl reaction volume using Platinum Taq High Fidelity polymerase (Thermo Fisher Scientific). The outer PCR reaction was performed on genomic DNA at the previously determined single-copy dilution using outer PCR primers BLOuterF (5′-AAA​TCT​CTA​GCA​GTG​GCG​CCC​GAA​CAG-3′) and BLOuterR (5′-TGA​GGG​ATC​TCT​AGT​TAC​CAG​AGT​C-3′). Touchdown cycling conditions were 94°C for 2 min and then 94°C for 30 s, 64°C for 30 s, and 68°C for 10 min for three cycles; 94°C for 30 s, 61°C for 30 s, and 68°C for 10 min for three cycles; 94°C for 30 s, 58°C for 30 s, and 68°C for 10 min for three cycles; 94°C for 30 s, 55°C for 30 s, and 68°C for 10 min for 41 cycles; and then 68°C for 10 min (Li et al., 2007; Ho et al., 2013).

### Q4PCR

Undiluted 1-µl aliquots of the NFL 1.PCR product were subjected to a Q4PCR reaction using a combination of four primer/probe sets that target conserved regions in the HIV-1 genome. Each primer/probe set consists of a forward and reverse primer pair as well as a fluorescently labeled internal hydrolysis probe as follows: PS: forward, 5′-TCT​CTC​GAC​GCA​GGA​CTC-3′; reverse, 5′-TCT​AGC​CTC​CGC​TAG​TCA​AA-3′; probe, 5′/Cy5/TTTGGCGTA/TAO/CTCACCAGTCGCC/3′/IAbRQSp (Integrated DNA Technologies; Bruner and Siliciano, 2018); *env*: forward, 5′-AGT​GGT​GCA​GAG​AGA​AAA​AAG​AGC-3′; reverse, 5′-GTC​TGG​CCT​GTA​CCG​TCA​GC-3′; probe, 5′/VIC/CCTTGGGTTCTTGGGA/3′/MGB (Thermo Fisher Scientific; Bruner et al., 2019); *gag*: forward, 5′-ATG​TTT​TCA​GCA​TTA​TCA​GAA​GGA-3′; reverse, 5′-TGC​TTG​ATG​TCC​CCC​CAC​T-3′; probe, 5′/6-FAM/CCACCCCAC/ZEN/AAGATTTAAACACCATGCTAA/3′/IABkFQ (Integrated DNA Technologies; Palmer et al., 2003); and *pol*: forward, 5′-GCA​CTT​TAA​ATT​TTC​CCA​TTA​GTC​CTA-3′; reverse, 5′-CAA​ATT​TCT​ACT​AAT​GCT​TTT​ATT​TTT​TC-3′; probe, 5′/NED/AAGCCAGGAATGGATGGCC/3′/MGB (Thermo Fisher Scientific; Schmid et al., 2010).

Each Q4PCR reaction was performed in a 10-µl total reaction volume containing 5 µl TaqMan Universal PCR Master Mix containing Rox (4304437; Applied Biosystems), 1 µl diluted genomic DNA, nuclease-free water, and the following primer and probe concentrations: PS, 675 nM of forward and reverse primers with 187.5 nM of PS internal probe; *env*, 90 nM of forward and reverse primers with 25 nM of *env* internal probe; *gag*, 337.5 nM of forward and reverse primers with 93.75 nM of *gag* internal probe; and *pol*, 675 nM of forward and reverse primers with 187.5 nM of *pol* internal probe. qPCR conditions were 94°C for 10 min, 40 cycles of 94°C for 15 s, and 60°C for 60 s. All qPCR reactions were performed in a 384-well plate format using the Applied Biosystem QuantStudio 6 Flex Real-Time PCR System.

### qPCR data analysis

We used QuantStudio Real-Time PCR Software version 1.3 (Thermo Fisher Scientific) for data analysis. The same baseline correction (start cycle: 3, end cycle: 10) and normalized reporter signal (ΔRn) threshold (ΔRn threshold = 0.025) was set manually for all targets/probes. Fluorescent signal above the threshold was used to determine the threshold cycle.

Samples with a threshold cycle value between 10 and 40 of any probe or probe combination were identified. Preliminary sequence analysis indicated that samples reacting with only a single primer/probe or only the PS+*gag* combination were defective, and these samples were mostly omitted from further analysis. All other samples showing reactivity with two or more of the four qPCR probes were selected for further processing.

### Nested NFL HIV-1 PCR (2.PCR)

The nested PCR reaction was performed on undiluted 1 µl aliquots of the NFL 1.PCR product. Reactions were performed in a 20 µl reaction volume using Platinum Taq High Fidelity polymerase (Thermo Fisher Scientific) and PCR primers 275F (5′-ACA​GGG​ACC​TGA​AAG​CGA​AAG-3′) and 280R (5′-CTA​GTT​ACC​AGA​GTC​ACA​CAA​CAG​ACG-3′; Ho et al., 2013) at a concentration of 800 nM. Touchdown cycling conditions were 94°C for 2 min and then 94°C for 30 s, 64°C for 30 s, and 68°C for 10 min for three cycles; 94°C for 30 s, 61°C for 30 s, and 68°C for 10 min for three cycles; 94°C for 30 s, 58°C for 30 s, and 68°C for 10 min for three cycles; 94°C for 30 s, 55°C for 30 s, and 68°C for 10 min for 41 cycles; and then 68°C for 10 min.

### Library preparation and sequencing

All nested PCR products were subjected to library preparation without prior gel visualization.

The Qubit 3.0 Fluorometer and Qubit dsDNA BR Assay Kit (Thermo Fisher Scientific) was used to measure DNA concentrations. Samples were diluted to a concentration of 10–20 ng/µl. Tagmentation reactions were performed using 1 µl of diluted DNA, 0.25 µl Nextera TDE1 Tagment DNA enzyme (catalog no. 15027865), and 1.25 µl TD Tagment DNA buffer (catalog no. 15027866; Illumina). Tagmented DNA was ligated to unique i5/i7 barcoded primer combinations using the Illumina Nextera XT Index Kit v2 and KAPA HiFi HotStart ReadyMix (2X; KAPA Biosystems) and then purified using AmPure Beads XP (Agencourt). 384 purified samples were pooled into one library and then subjected to paired-end sequencing using Illumina MiSeq Nano 300 V2 cycle kits (Illumina) at a concentration of 12 pM.

### Sequence assembly

HIV sequence reconstruction was performed by our in-house pipeline (HIV Assembler), a pipeline for the assembly of raw sequencing reads into annotated HIV genomes, capable of reconstructing thousands of genomes within hours. First, we used a quality-control check to trim Illumina adapters and low-quality bases followed by multiple assembly steps that combine two widely used classes of algorithms, de Bruijn graph and overlap layout consensus. de Bruijn graph is performed by SPAdes for initial de novo assembly of contigs which are aligned via BLAST to a database of HIV genome sequences to select the closest reference. Overlap layout consensus is performed by MIRA (Mimicking Intelligent Read Assembly) v4.0.2 in two steps. First, a modified version of the closest reference is generated by alignment to the contigs produced by SPAdes genome assembler v3.9.0. After, the modified reference is used as a scaffold for the final reference-guided assembly of the initial trimmed reads. Finally, the HIV genome sequence is annotated by alignment to HXB2 using ClustalW. Sequences with double peaks (cutoff consensus identity for any residue <75%) or limited reads (empty wells ≤500 sequencing reads) were omitted from downstream analyses. The HIV Assembler is constructed with Snakemake, a workflow management system, allowing reproducible data analyses and scalability to cluster and cloud environments.

### Data availability

Proviral sequences have been deposited in GenBank with the accession nos. MN090187–MN090943.

### Phylogenetic analysis

Nucleotide alignments of intact *env* sequences were translation aligned using ClustalW v.2.148 under the BLOSUM cost matrix. Sequences with premature stop codons and frameshift mutations that fell in the gp120 surface glycoprotein region were excluded from all analyses. Maximum likelihood phylogenetic trees were then generated from these alignments with RAxML v.8.2.950 under the GTRGAMMA model with 1,000 bootstraps.

### Euler diagrams

Identical *env* sequences captured by each method (Q^2^VOA, NFL, and Q4PRC) were considered as shared sequences. The number inside overlapping areas in the Euler diagram is the sum of all shared sequences. The fraction of shared sequence from individual methods is shown in Table S3.

### Statistical analyses

Statistical analyses were performed using GraphPad Prism 7.0d for Mac OS X.

### Online supplemental material

Fig. S1 shows the predicted detection of HIV-1 clades A and C intact proviral sequences. Fig. S2 shows the sequence classification process. Fig. S3 shows the intra assay variability and the number of CD4^+^ T cells tested in Q^2^VOA, NFL sequencing, and Q4PCR. Fig. S4 shows phylogenetic trees of *env* sequences. Fig. S5 shows the analysis of single-nucleotide polymorphisms. Table S1 shows the quantitative analysis of Q4PCR. Table S2 shows the quantitative analysis of intact proviruses. Table S3 shows identical *env* sequences obtained with Q^2^VOA, NFL, and Q4PCR. Table S4 shows the qualitative sequence analysis. Table S5 shows the Q4PCR probe analysis. Table S6 shows the Q4PCR probe analysis for individual participants.

## Supplementary Material

Supplemental Material (PDF)

Table S1 (Excel)

Table S2 (Excel)

Table S3 (Excel)

Table S4 (Text)

Table S5 (Excel)

Table S6 (Excel)
