# Supplementary material for: Combination of quadruplex qPCR and next-generation sequencing for qualitative and quantitative analysis of the HIV-1 latent reservoir
Source: J Exp Med. 2019 Jul 26;216(10):2253–64. doi: 10.1084/jem.20190896 (PMC6781006; doi:10.1084/jem.20190896)
Supplement: Supplemental Material (PDF) [file JEM_20190896_sm.pdf]

Supplemental material

Gaebler et al., https://doi.org/10.1084/jem.20190896

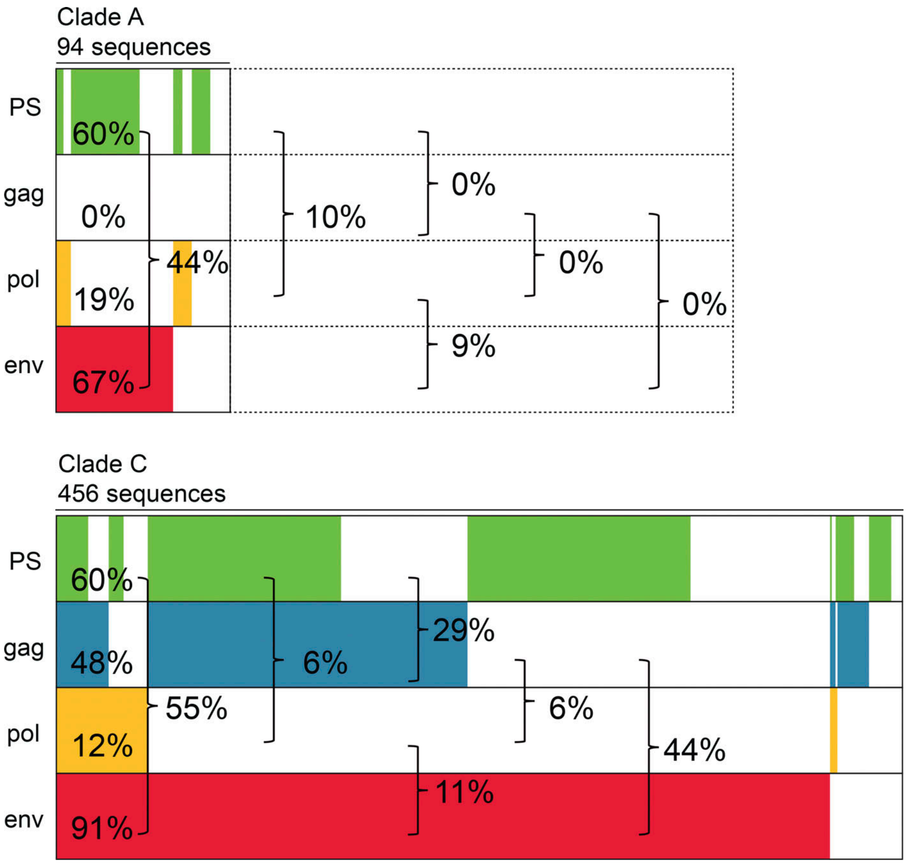

Figure S1. **Predicted detection of HIV-1 clades A and C.** Predicted detection of 94 intact clade A and 456 clade C proviral sequences from the Los Alamos HIV sequence database by primer/probe sets that target PS (green), *gag* (blue), *pol* (yellow), and *env* (red) regions. Predicted signals are represented by the presence of the color of the respective primer/probe set. Sequences containing polymorphisms that prevent signal detection are represented by the absence of color. The percentage indicates the fraction of detected sequences for individual primer/probe sets or combinations of two primer/probe sets (brackets).

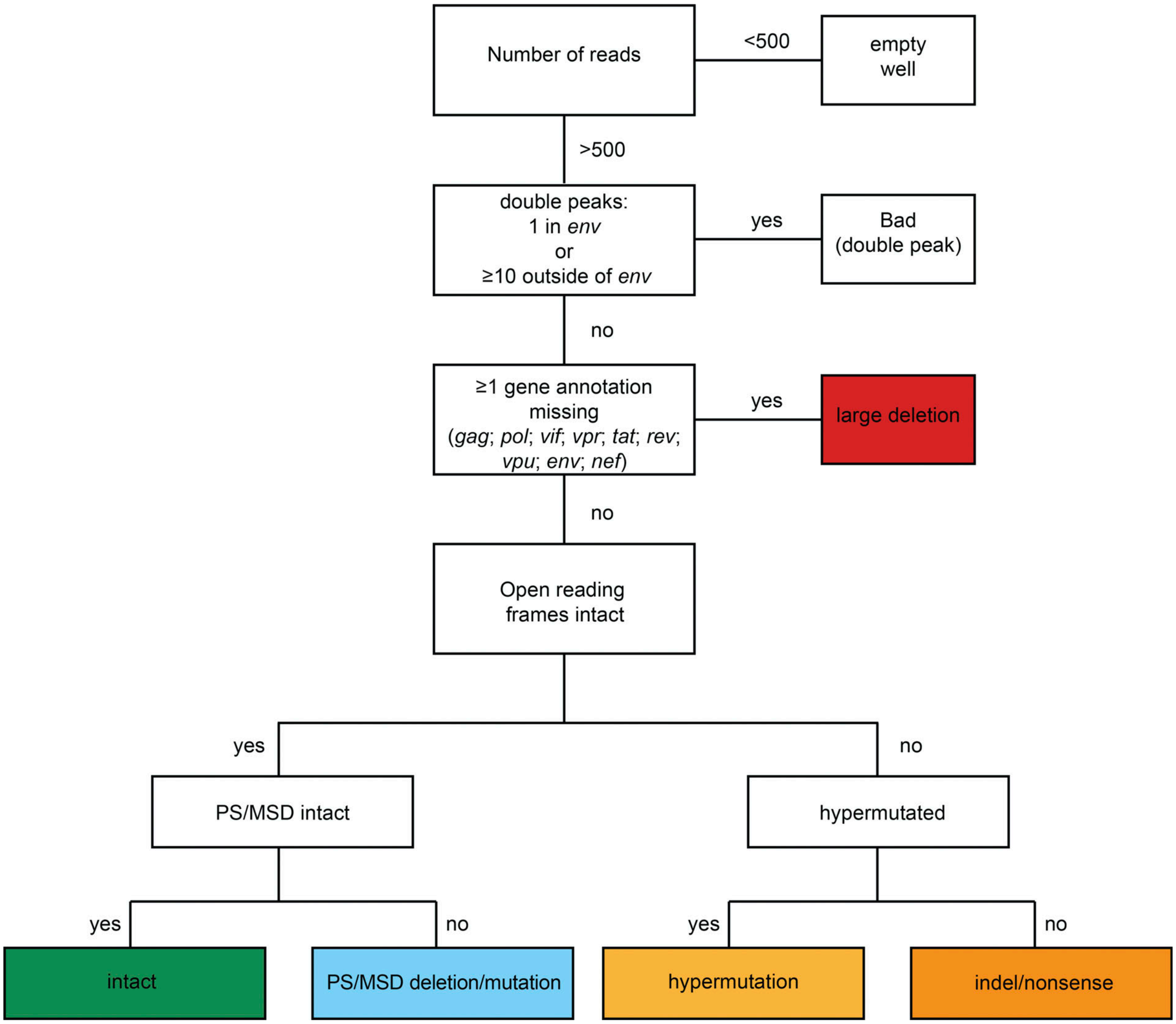

Figure S2. **Sequence classification process.** Sequences with double peaks (cutoff consensus identity for any residue <75%) or limited reads (empty wells ≤500 sequencing reads) were omitted from downstream analyses. Assembled HIV genomes were annotated by alignment to HXB2 to identify sequences with missing gene annotations (large deletions), premature stop codons, out-of-frame insertions or deletions (hypermutation or indel), or PS and the MSD site deletions and mutations.

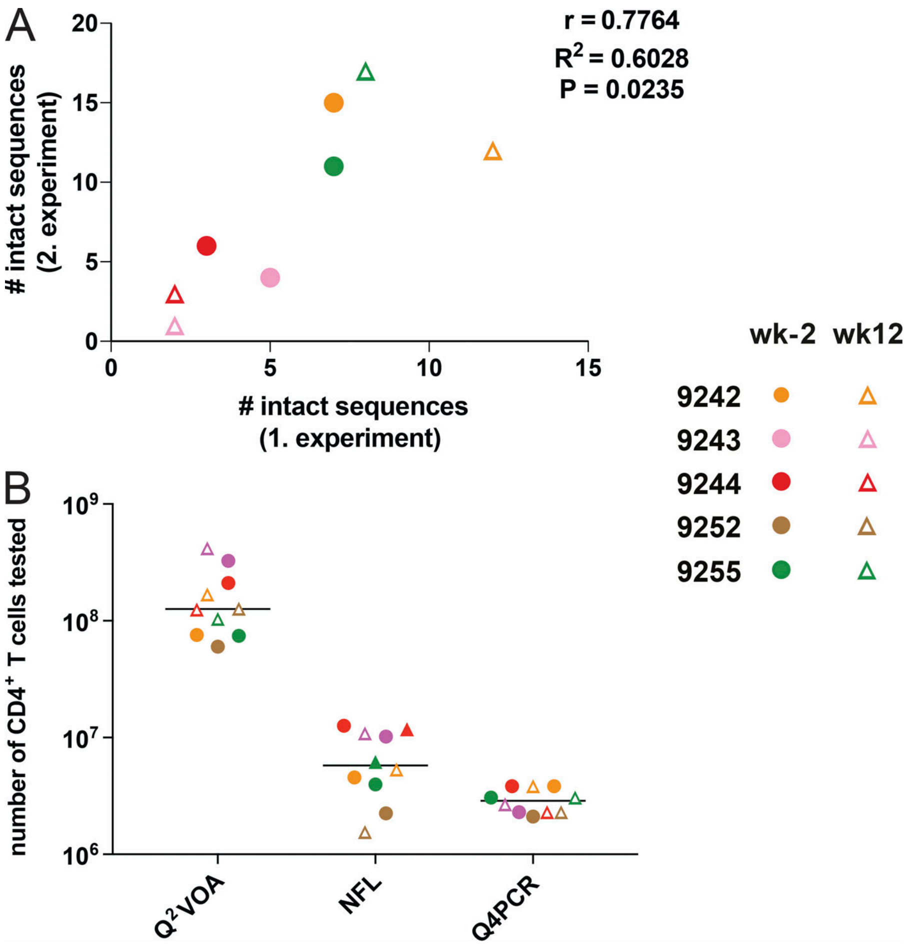

Figure S3. **Assay variability and number of CD4⁺ T cells tested. (A)** Pearson correlation between the number of intact proviruses identified in two sets of independent experiments from four individuals at preinfusion (wk−2; circles) and week 12 (triangles) time points. **(B)** Scatter plot showing the number of CD4⁺ T cells tested in Q²VOA, NFL sequencing, and Q4PCR at preinfusion (wk−2; circles) and week 12 (triangles) time points. Horizontal bars indicate median values.

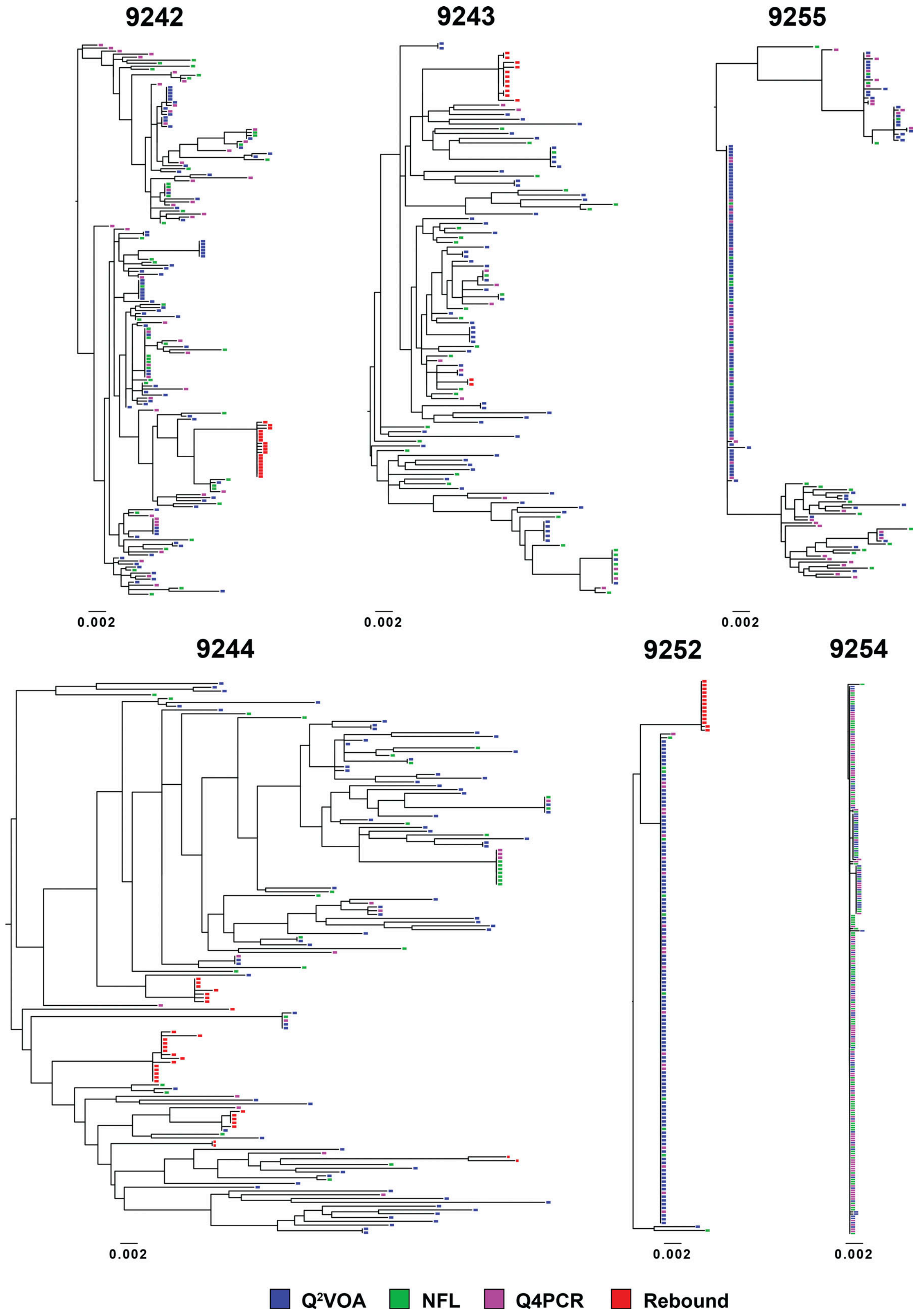

Figure S4. **Phylogenetic trees of *env* sequences.** Maximum likelihood phylogenetic trees of *env* sequences obtained with Q²VOA (blue), NFL sequencing (green), Q4PCR (purple), and rebound plasma SGA or outgrowth cultures (red). Sequences from the preinfusion and week 12 time points were combined for Q²VOA, NFL sequencing, and Q4PCR.

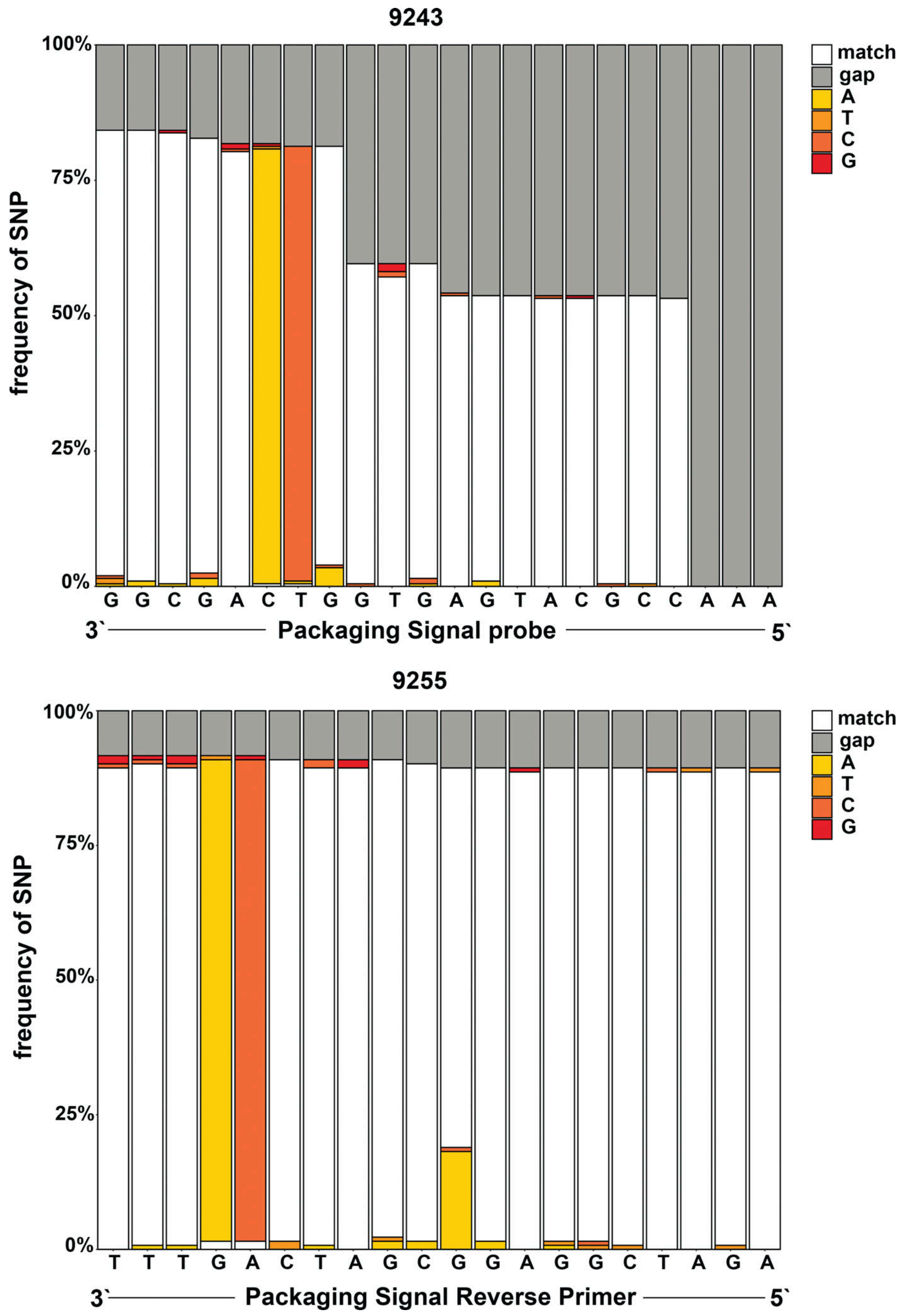

Figure S5. **Single-nucleotide polymorphism analysis.** Stacked bar graphs depicting sequence identity obtained by aligning all (defective and intact) proviral sequences from patients 9243 and 9255 with PS probe and PS reverse primer, respectively. The frequency of identical nucleotides is shown in white (match). The frequencies of single-nucleotide polymorphisms (SNPs) that lead to mismatches with tested primer/probe are depicted in yellow (adenosine), orange (thymine), light red (cytosine), dark red (guanine), and gray (gap in alignment). The PS probe and reverse primer are depicted in reverse-complement orientation.

Tables S1–S6 are provided online as separate Excel files. Table S1 shows quantitative analysis of Q4PCR. Table S2 shows quantitative analysis of intact proviruses. Table S3 lists identical *env* sequences obtained with Q²VOA, NFL, and Q4PCR. Table S4 shows qualitative analysis. Table S5 shows probe analysis. Table S6 shows probe analysis for individual participants.
